# Exploring the Role of Stress Rumination in Caregiver Cognitive Performance

**DOI:** 10.1093/geroni/igaf122.1256

**Published:** 2025-12-31

**Authors:** Kate Singer

**Affiliations:** Miami University, Oxford, Ohio, United States

## Abstract

Caregiving is a rewarding and strenuous activity affecting physiological, psychological, and cognitive processes. While caregiving stress has been studied previously, these studies have overlooked rumination and its effect on cognition. The present study explored how recalling stressful caregiving experiences affected caregiver stress levels and executive functions using the AX-CPT. Neural correlates underlying cognitive performance were also examined using functional near-infrared spectroscopy (fNIRS). 36 caregivers were recruited for this study, including both formal and informal caregivers of persons diagnosed with a variety of chronic conditions. Our findings indicated that longer lifetime durations of caregiving activities, not recalling stressful experiences, were associated with poorer performance on the AX-CPT in the present sample of caregivers, though no changes in neural correlates were observed. Future studies should continue to examine the impact of momentary stress recall on caregiver outcomes and the role of caregiving duration their executive functions.

